# Supplementary material for: Interplay of hidden orbital order and superconductivity in CeCoIn5
Source: Nat Commun. 2023 May 24;14:2984. doi: 10.1038/s41467-023-38760-7 (PMC10209141; doi:10.1038/s41467-023-38760-7)
Supplement: Supplementary file 1 — Supplementary Information [file 41467_2023_38760_MOESM1_ESM.pdf]

Supplementary information for

Interplay of Hidden Orbital Order and Superconductivity in $CeCoIn_5$

Weijiong Chen, Clara Neerup Breiø, Freek Massee, M.P. Allan, C. Petrovic, J.C. Séamus Davis, P.J. Hirschfeld, B.M. Andersen and Andreas Kreisel

1. Theoretical Model of (π,π) Orbital Order.

Our minimal 2D square-lattice model Hamiltonian $\mathcal{H}_0(\mathbf{k})$ includes only electronic states of d_{xz}/d_{yz}-orbitals on each lattice point. The assumed band structure is similar to the one used in Ref. 1. We enlarge the unit cell to a two-ion basis allowing for the incorporation of a staggered, nematic orbital order ((π,π) orbital order) preserving the translational and global rotational symmetries as it can be seen in Fig. 1a. Defining the two-ion(sublattice), two-orbital basis as

$\boldsymbol{c}_{\mathbf{k},\sigma} = \left(c_{A,xz,\sigma}(\mathbf{k}), c_{A,yz,\sigma}(\mathbf{k}), c_{B,xz,\sigma}(\mathbf{k}), c_{B,yz,\sigma}(\mathbf{k})\right)^T$, where $c_{\nu,\mu}(\mathbf{k})$ annihilates an electron in orbital d_μ on sublattice ν with momentum $\boldsymbol{k}$ and spin σ, we can write

$$\mathcal{H}_0(\mathbf{k}) = \begin{pmatrix} \epsilon_3(\mathbf{k}) - \mu & \epsilon_4(\mathbf{k}) & \epsilon_1(\mathbf{k}) + \epsilon_2(\mathbf{k}) & 0 \\ \epsilon_4(\mathbf{k}) & \epsilon_3(\mathbf{k}) - \mu & 0 & \epsilon_1(\mathbf{k}) - \epsilon_2(\mathbf{k}) \\ \epsilon_1^*(\mathbf{k}) + \epsilon_2^*(\mathbf{k}) & 0 & \epsilon_3(\mathbf{k}) - \mu & \epsilon_4(\mathbf{k}) \\ 0 & \epsilon_1^*(\mathbf{k}) - \epsilon_2^*(\mathbf{k}) & \epsilon_4(\mathbf{k}) & \epsilon_3(\mathbf{k}) - \mu \end{pmatrix} \quad \text{(S1)}$$

with

$$\epsilon_1(\mathbf{k}) = -\frac{1}{2}(t_1 + t_2)\left(1 + e^{ik_x} + e^{-ik_y} + e^{i(k_x - k_y)}\right) \quad \text{(S2)}$$

$$\epsilon_2(\mathbf{k}) = -\frac{1}{2}(t_1 - t_2)\left(1 - e^{ik_x} - e^{-ik_y} + e^{i(k_x - k_y)}\right) \quad \text{(S3)}$$

$$\epsilon_3(\mathbf{k}) = -2t_3\left(cos(k_x) + cos(k_y)\right) \quad \text{(S4)}$$

$$\epsilon_4(\mathbf{k}) = -2t_4\left(cos(k_x) - cos(k_y)\right) \quad \text{(S5)}$$

We adopt the hopping parameters $\{t_1, t_2, t_3, t_4\} = \{-1.0, 1.3, -0.85, -0.85\}$ from Ref. 1 and set the chemical potential $\mu = 2.25|t_1|$ in all computations. In this basis, the (π,π) orbital order can formally be written as

$$\mathcal{H}_{oo}(\boldsymbol{k}) = \Delta_{oo}\, \boldsymbol{c}^\dagger_{\mathbf{k},\sigma}\, s_3 \sigma_3\, \boldsymbol{c}_{\mathbf{k},\sigma} \quad \text{(S6)}$$

where s_i and σ_i ($i = 1,2,3$) are the Pauli matrices in sublattice and orbital space, respectively, and $\Delta_{oo} = 0.25|t_1|$ is the orbital order parameter. Note that because of the s_3 in (S6), exchanging the sign of Δ_{oo} amounts to interchanging the sublattices.

To include superconductivity of $d_{x^2-y^2}$-symmetry in a multi-orbital setting, we follow the work performed by Graser *et al.* in Ref. 1, where the real space pairings arising from spin-fluctuations in the basis of the two relevant orbitals are computed. In the single-ion unit cell picture, the largest pairing amplitudes are the two nearest-neighbor (NN) bonds along the y-axis (x-axis) for the d_{xz}-orbital (d_{yz}-orbital) as well as all four NNN bonds in both orbitals. Interorbital pairings are negligible. Rewriting these six pairing terms of each orbital channel in momentum space and setting these identical on the sublattices yields

$$\begin{aligned}\Delta_{\mathrm{d}}(\mathbf{k}) = -\Delta_1 \Big(& (e^{ik_x} + e^{-ik_y}) c^\dagger_{A,xz,\uparrow}(\mathbf{k}) c^\dagger_{B,xz,\downarrow}(-\mathbf{k}) + (e^{-ik_x} + e^{ik_y}) c^\dagger_{B,xz,\uparrow}(\mathbf{k}) c^\dagger_{A,xz,\downarrow}(-\mathbf{k}) \\ & - (1 + e^{i(k_x-k_y)}) c^\dagger_{A,yz,\uparrow}(\mathbf{k}) c^\dagger_{B,yz,\downarrow}(-\mathbf{k}) \\ & - (1 + e^{-i(k_x-k_y)}) c^\dagger_{B,yz,\uparrow}(\mathbf{k}) c^\dagger_{A,yz,\downarrow}(-\mathbf{k}) \Big) \\ & - 2\Delta_2 \Big(cos(k_x) \\ & + cos(k_y) \Big) \sum_{\nu=A,B} \left(c^\dagger_{\nu,xz,\uparrow}(\mathbf{k}) c^\dagger_{\nu,xz,\downarrow}(-\mathbf{k}) - c^\dagger_{\nu,yz,\uparrow}(\mathbf{k}) c^\dagger_{\nu,yz,\downarrow}(-\mathbf{k}) \right)\end{aligned} \tag{S7}$$

where $\{\Delta_1, \Delta_2\} = \{0.025, 0.02\}$ is the pairing amplitude between first and second neighbors, respectively, and the spin-singlet structure is implicit. In this model, $\Delta_{\mathrm{oo}} \sim 3\Delta_{\mathrm{d}}$, is estimated from the experimental fact that orbital order on the surface of $CeCoIn_5$ exists even at 6 K while the superconducting temperature of $CeCoIn_5$ is 2.3K. We also calculate the anisotropy for $\Delta_{\mathrm{oo}} = 0.1\,|t_1| \sim \Delta_{\mathrm{d}}$ (Fig. S8). The results are qualitatively identical to the results shown in Fig. S4 for $\Delta_{\mathrm{oo}} = 0.25\,|t_1|$.

Defining the Nambu spinor as

$$\psi_{\mathbf{k}} = (\boldsymbol{c}_{\mathbf{k},\uparrow}, \boldsymbol{c}^\dagger{}_{-\mathbf{k},\downarrow}) = \left(c_{A,xz,\uparrow}(\mathbf{k}), c_{A,yz,\uparrow}(\mathbf{k}), c_{B,xz,\uparrow}(\boldsymbol{k}), c_{B,yz,\uparrow}(\mathbf{k}), c^\dagger_{A,xz,\downarrow}(-\mathbf{k}), c^\dagger_{A,yz,\downarrow}(-\mathbf{k}), c^\dagger_{B,xz,\downarrow}(-\mathbf{k}), c^\dagger_{B,yz,\downarrow}(-\mathbf{k}) \right)^T \tag{S8}$$

and neglecting the spin degree of freedom, we can write the full Hamiltonian as

$$H = \sum_{\mathbf{k}} \psi^\dagger_{\mathbf{k}} \begin{pmatrix} \mathcal{H}_0(\mathbf{k}) + \mathcal{H}_{oo}(\mathbf{k}) & \Delta_{\mathrm{d}}(\mathbf{k}) \\ \Delta^\dagger_{\mathrm{d}}(\mathbf{k}) & -\mathcal{H}^*_0(-\mathbf{k}) - \mathcal{H}^*_{oo}(-\mathbf{k}) \end{pmatrix} \psi_{\mathbf{k}} \tag{S9}$$

This minimal model Hamiltonian only includes the key ingredients of the orbital order, Co d_{xz}/d_{yz} orbits.

In this work, we only consider the simplest model Hamiltonian including staggered orbital order and it is not identical to the real Fermi surface of $CeCoIn_5$. We do not discuss a more complete model including both Ce and In atoms and the superconductivity originating from Ce atoms, since such issues are both beyond the scope of our current work and not relevant to its conclusions. Nevertheless, as shown in Fig. S7, the overall pattern of the real part of BQPI is still present in a good agreement between the calculation and the experiment except some inconsistencies in the exact period of the Friedel oscillations. This implies that our model indeed captures the key ingredients of symmetry-breaking QPI induced by the orbital order.

2. Quasiparticle Interference Simulation of Two Sublattice Scatterings

The local density of states (LDOS) is computed using a T-matrix approximation as

$$N(\mathbf{R},\gamma,E) = -\frac{1}{\pi}\mathrm{Im}\big(G^R(\mathbf{0},E) + G^R(\mathbf{R},E)T(\mathbf{0},E)G^R(-\mathbf{R},E)\big)_{\gamma\gamma} \quad \text{(S10)}$$

where $\boldsymbol{R}$ is the real-space position of the two-ion unit cell, $\gamma \in \{\nu = A,B\,;\,\mu = xz,yz\}$, the T-matrix is given by $T(\mathbf{0},\mathrm{E}) = \left[1 - H_{\mathrm{imp}}(\mathbf{0})G^{\mathbf{R}}(\mathbf{0},\mathrm{E})\right]^{-1}H_{\mathrm{imp}}(\mathbf{0})$ with

$$H_{\mathrm{imp}}(\mathbf{0}) = V_{\mathrm{imp}}\,\psi_{\mathbf{0}}^{\dagger}\,\tau_3\frac{1}{2}(s_0 \pm s_3)\sigma_0\,\psi_{\mathbf{0}} = V_{imp}\sum_{\mathbf{k},\mathbf{k}'}\psi_{\mathbf{k}}^{\dagger}\,\tau_3\frac{1}{2}(s_0 \pm s_3)\sigma_0\,\psi_{\mathbf{k}'} \quad \text{(S11)}$$

where τ_i ($\mathrm{i} = 1,2,3$) are the Pauli matrices in Nambu space, s_0 and σ_0 are identity matrices in sublattice and orbital space, respectively, and the sign refers to the impurity position at sublattice $\boldsymbol{a}\,(+)$ or $\boldsymbol{b}\,(-)$, and $G^R(\mathbf{R},\mathrm{E}) = \mathcal{G}^0(\mathbf{R},i\omega_n \to \mathrm{E} + i\eta) = \sum_{\mathbf{k}} e^{i\boldsymbol{k}\mathbf{R}}\mathcal{G}^0(\mathbf{k},i\omega_n)$. We set $V_{\mathrm{imp}} = 10|t_1|$ and $\eta = 0.001|t_1|$ in all simulations. The sublattice site resolved local density of state (LDOS), $N(\tilde{\mathbf{r}},\boldsymbol{E})$, is uniquely mapped from the set $\{\mathbf{R},\nu\}$.

To improve the direct comparison to experiment we implement two modifications to the calculated LDOS. First, we take into account that the tunnelling process to the STM tip is in the exponential limit, i.e. the STM tip is several Å above the surface and the tunnelling conductance is proportional to the local density of states at that position. For our model, we use extended, atomic-like orbitals to calculate the local density of states[2,3]

$$N(\mathbf{r},E) = \sum_{\tilde{\mathbf{r}},\mu} N(\tilde{\mathbf{r}},\mu,E)\left|w_{\tilde{\mathbf{r}},\mu}(\mathbf{r})\right|^2 \quad \text{(S12)}$$

where $w_{\tilde{\mathbf{r}},\mathrm{xz(yz)}}(\mathbf{r}) = \frac{xz(yz)}{r}e^{-\alpha r}$ are hydrogen-like $d_{xz(yz)}$ orbitals and $\mu = xz,yz$. Note that we perform the $\tilde{\mathbf{r}}$-summation over a 5x5 grid and this approximation neglects off-

diagonal and non-local contributions of the lattice Green function which are expected to be small[2]. The vector $\mathbf{r} = (\mathbf{x}, \mathbf{y}, \mathbf{z})$ is defined on this 5x5 grid for each atomic site and we use the parameters $\{z, \alpha\} = \{1.05a_0, 1/a_0\}$. Second, to account for the finite energy resolution in experimental data, we introduce a Gaussian energy convolution

$$N(\boldsymbol{r}, E) = \sum_{E'} N(\boldsymbol{r}, E') f(E - E', \sigma) \tag{S13}$$

with $f(E - E', \sigma) = \frac{1}{\sigma\sqrt{2\pi}} e^{-\frac{1}{2}\left(\frac{E'-E}{\sigma}\right)^2}$. $\sigma = \Delta/12$ corresponds to the experimental energy resolution $\sim$100meV. Fig. S1 gives the direct comparison between calculated LDOS $N(E)$ and measured density of state $g(E)$ far from the impurities and at the impurity/defect site to reveal that our choice of model parameters allows to describe the spectral properties of the impurities in the experiment. In Fig. S2 we present the anisotropy in real space (see main text) as calculated in the superconducting state showing that the anisotropy at zero energy (E=0) is much larger than at a finite energy above the superconducting gap. In contrast, Fig. S3 shows the corresponding result as obtained without superconducting order (normal state) where the anisotropy is very small for both E = 0 and at finite energy. As a consequence, the orbital order would be difficult to detect in this local probe. The same conclusions can be read off from Fig. S4 where the anisotropy is plotted as function of energy with and without superconducting order.

We point out that the authors of Ref. [4] report a similar symmetry-breaking QPI caused by the nematicity in the FeSeTe system. Their observation is distinct from our result in two aspects. First, the global QPI analysis they perform discovers the order that breaks overall crystal lattice symmetry but should not yield anti-ferro-orbital order which perserves the global C_4 symmetry as in $CeCoIn_5$. Second, the nematicity they discover is only observed in the non-superconductive state at high energy beyond the coherence peak. This is also distinct from our present picture where the anisotropy from orbital order is significantly enhanced within the superconductive quasiparticles below the superconducting gap.

3. Multi-atom Technique

The multi-atom technique[5] is overlapping and averaging the same type of defects to suppress the random noise and highlight the common features of the defects. we first identify the coordinates $\mathbf{R}_i$ of the centers of the defects from the topography. The defects

are chosen in the topography by eye selecting only those of the same type as in Fig. 3 and 4, since they are well allocated at sublattice site a/b. Then, the selected defects at sublattice a/b are distinguished by the surrounding scattering pattern in $g(\mathbf{q}, E=0)$ map.

Here we choose one defect as example. The chosen defects are marked by a red circle in Fig. 5a,e (topography) and 5b,f ($g(\mathbf{q}, E=0)$). The exact coordinate $\mathbf{R}_i = (x_i, y_i)$ for the center of the defect is figured out by calculating the center of mass of with intensity as the weights in the subsidence region around the impurity as seen in the topography. The shift operation is done in the $\mathbf{q}$-space on Fourier transformed map $T(\mathbf{q})$ and $g(\mathbf{q}, E)$ by

$$T_i^S(\mathbf{q}) = e^{i\mathbf{q}\cdot\mathbf{R}_i} T(\mathbf{q}) \tag{S14}$$

$$g_i^S(\mathbf{q}, E) = e^{i\mathbf{q}\cdot\mathbf{R}_i} g(\mathbf{q}, E) \tag{S15}$$

This step executes a shift with periodic boundary conditions. After the inverse Fourier transformation, the shifted data are shown in Fig. S6b,f. The defect is shifted to the center of the map. Finally, we overlap and average the shifted $g_i^S(\mathbf{r}, E)$ of all defects and get MA-averaged image $g_{MA}(\mathbf{r}, E)$.

$$T_{MA}(\mathbf{r}) = \frac{\sum_{i=1}^{N} T_i^S(\mathbf{r})}{N} \tag{S16}$$

$$g_{MA}(\mathbf{r}, E) = \sum_{i=1}^{N} g_i^S(\mathbf{r}, E)/N \tag{S17}$$

$T_{MA}(\mathbf{r})$ and $g_{MA}(\mathbf{r}, E)$ at sublattice a(b) are shown in Fig. S6c,g(d,h).

4. Identification of Termination Surface

As reported by Ref. [6], three different cleaved surfaces can be found in $CeCoIn_5$: Co surface, CeIn surface and In_2 surface. We first rule out the In_2 surface because this surface is reconstructed. Both Co surface and CeIn surface show the same lattice constant ~4.6Å. Here, we identify our measured surface as Co surface by two observations.

First, Fig. S8 shows the measured scanning tunneling spectrum on our sample surface. The spectrum presents a dip at ~5meV corresponding to the heavy-fermion hybridization gap. This spectrum is exactly the same as the spectrum measured on Co surface in Ref. [7], except that our energy resolution is better. However, the spectrum of the CeIn surface in Ref. [7] displays that the density of states at -30meV is larger than that at 30meV, different from what we observed.

Second, since the orbital order breaks the equivalence of Co sites in sublattice ***a*** and ***b***, two degenerate states should appear on the surface. At the interface of these two degenerate states, domain boundaries should form. Ref. [7] reports that the domain boundaries only appear on Co surface, implying that the orbital order occurs only on Co surface. We also observe many domain boundaries on our measured surface (Fig. S9), indicating our cleaved surface is Co-terminated.

Furthermore, Fig. S10 shows two nearby domains with several defects close to the domain boundary. In the $g(\mathbf{r}, E = 0)$ map (Fig. S10c) in the same field of view in Fig. S10a, we choose the same type of defects as in Figs. 3 and 5 (of the main text), which apparently break the local C_4 symmetry in the superconducting state at E=0, two in the left domain (domain 1) and one in the right domain (domain 2). According to their local anisotropy, we can distinguish at which sublattice sites these defects are located, and extract the sublattice ***a/b*** site order in each domain (red and blue dot in Fig. S10d). On the other hand, in Fig. S10b, the arrangement of Co atoms near the domain boundary can be directly visualized after we adjusted the colormap limits. Finally, in Fig. S10d, we draw a schematic diagram of sublattice ***a/b*** orders near the domain boundary, combining both the arrangement of the atoms shown in Fig. S10b and the sublattice ***a/b*** site order in each domain extracted by Fig.S10c. It clearly shows that the sublattice ***a/b*** site order in the two domains are opposite. This confirms that the domain boundary indeed forms at the interface of two degenerate orbital order states.

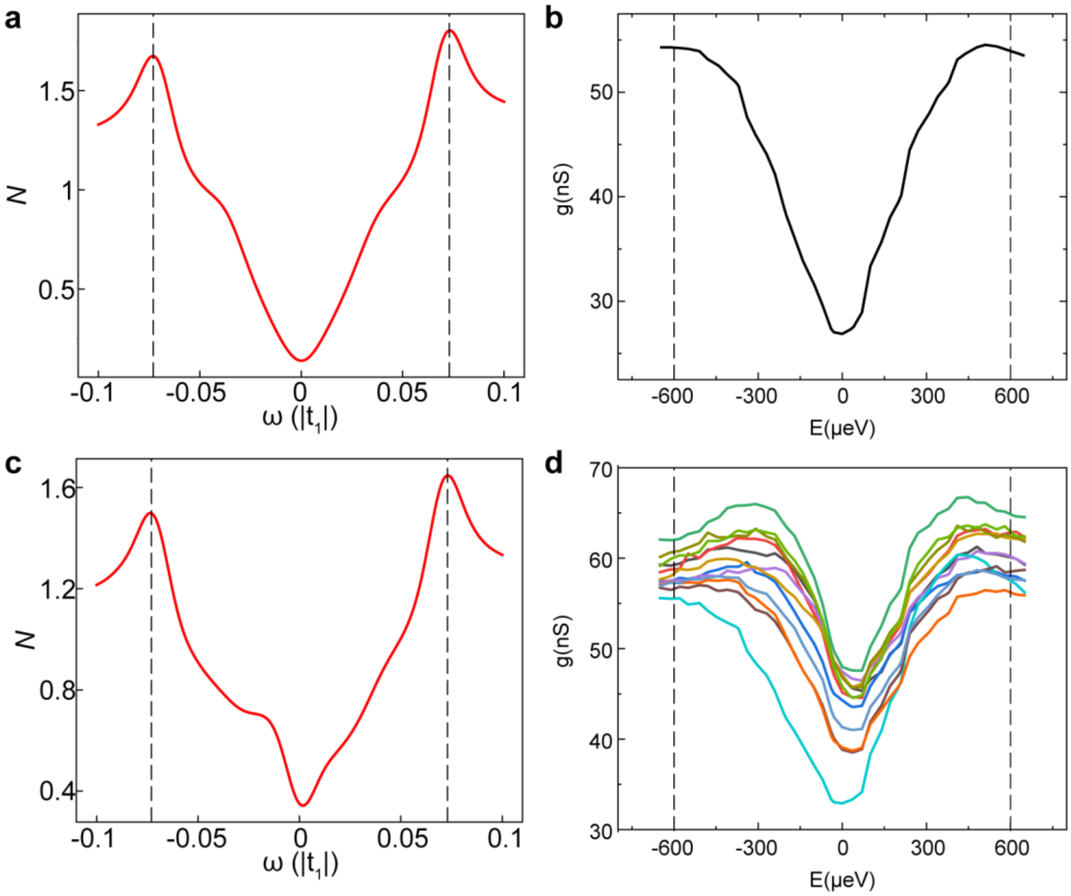

Figure S1. Simulated and measured spectra on the surface of $CeCoIn_5$

a,b Homogeneous spectra showing the V-shape signature of a $d_{x^2-y^2}$ superconducting gap obtained from the simulation of our model with V_{imp} = 0.0 (**a**) and measurement (**b**) at a site far from any impurities.

c,d Simulated (**c**) and measured (**d**) spectra obtained at the impurity site positioned at either sublattice ***a*** or ***b***.

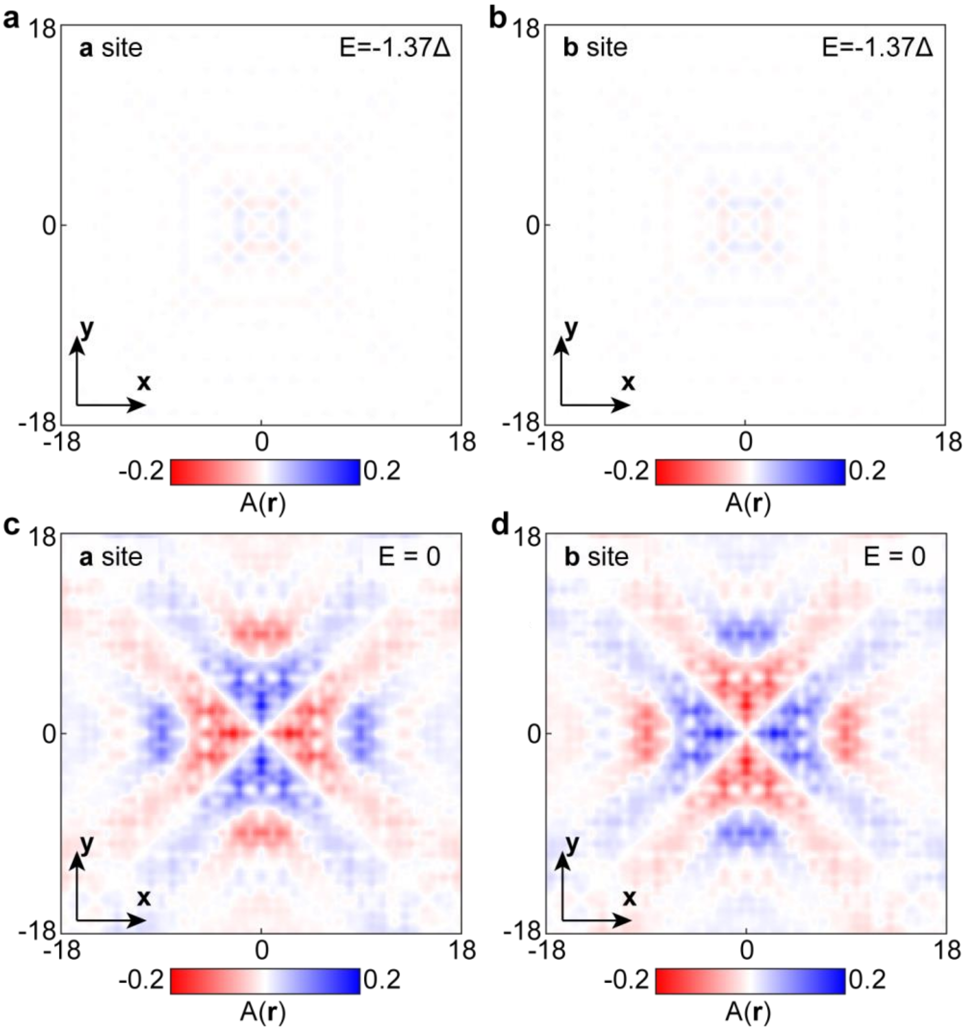

Figure S2. Local anisotropy obtained from the theoretical model

a,b The local anisotropy $A(\mathbf{r},\ E)$ with the impurity atom at sublattice ***a*** (**a**) and sublattice ***b*** (**b**) at the energy well above the superconducting gap $E > |\Delta|$. The local anisotropy $A(\mathbf{r},\ E)$ is no more than 2%.

c,d The local anisotropy $A(\mathbf{r},\ E)$ with the impurity atom at sublattice ***a*** (**c**) and sublattice ***b*** (**d**) at the energy well below the superconducting gap $E < |\Delta|$. Identical to Fig. 2c,f. The local anisotropy $A(\mathbf{r},\ E)$ approaches 20%.

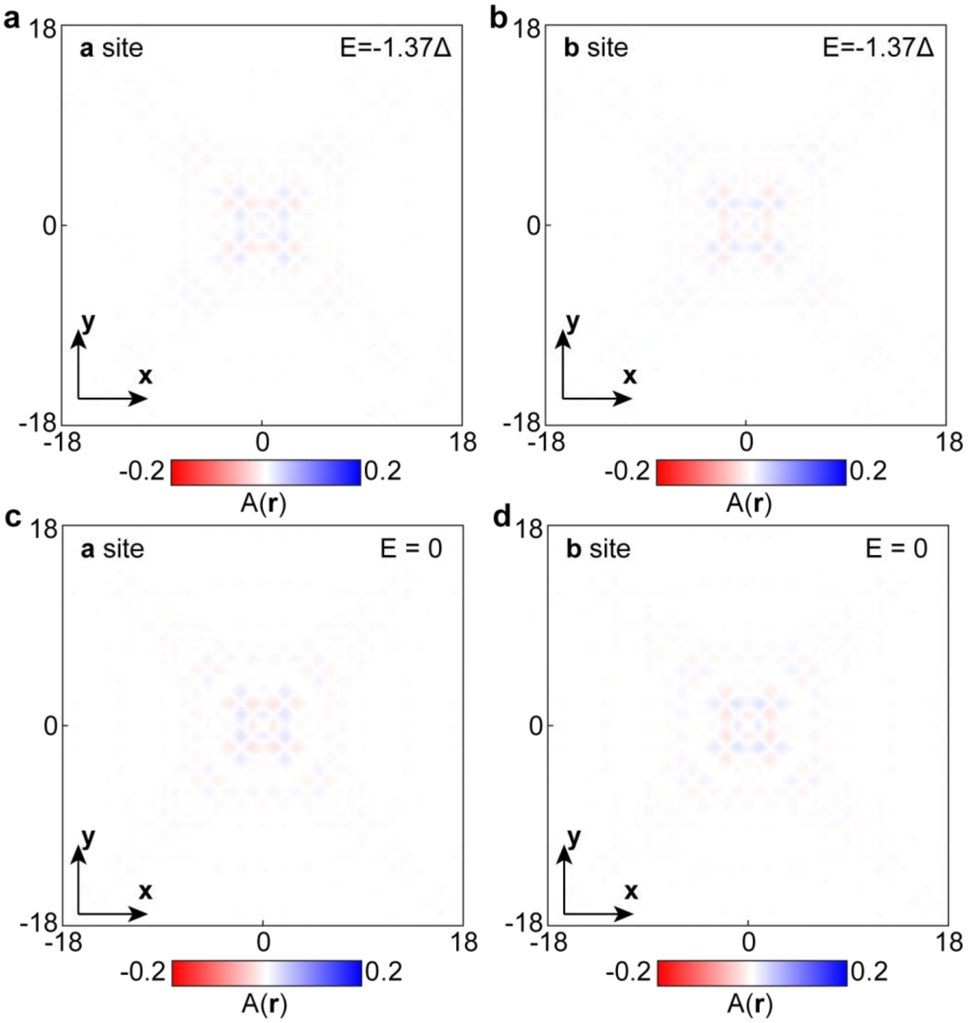

Figure S3. Local anisotropy obtained from the theoretical model in the normal state.

a,b The local anisotropy $A(\mathbf{r},\ E)$ in the normal state with the impurity atom at sublattice $\boldsymbol{a}$ (**a**) and sublattice $\boldsymbol{b}$ (**b**) at $E > |\Delta|$. Simulations computed by setting $\{\Delta_1, \Delta_2\} = \{0.0,\ 0.0\}$.

c,d The local anisotropy $A(\mathbf{r},\ E)$ in the normal state with the impurity atom at sublattice $\boldsymbol{a}$ (**c**) and sublattice $\boldsymbol{b}$ (**d**) at $E = 0$. Simulations computed by setting $\{\Delta_1, \Delta_2\} = \{0.0,\ 0.0\}$. The local anisotropy $A(\mathbf{r},\ E)$ is less than 2% at both energies, similar to the simulation of the superconducting state at $E > |\Delta|$ (Fig. S2 a,b).

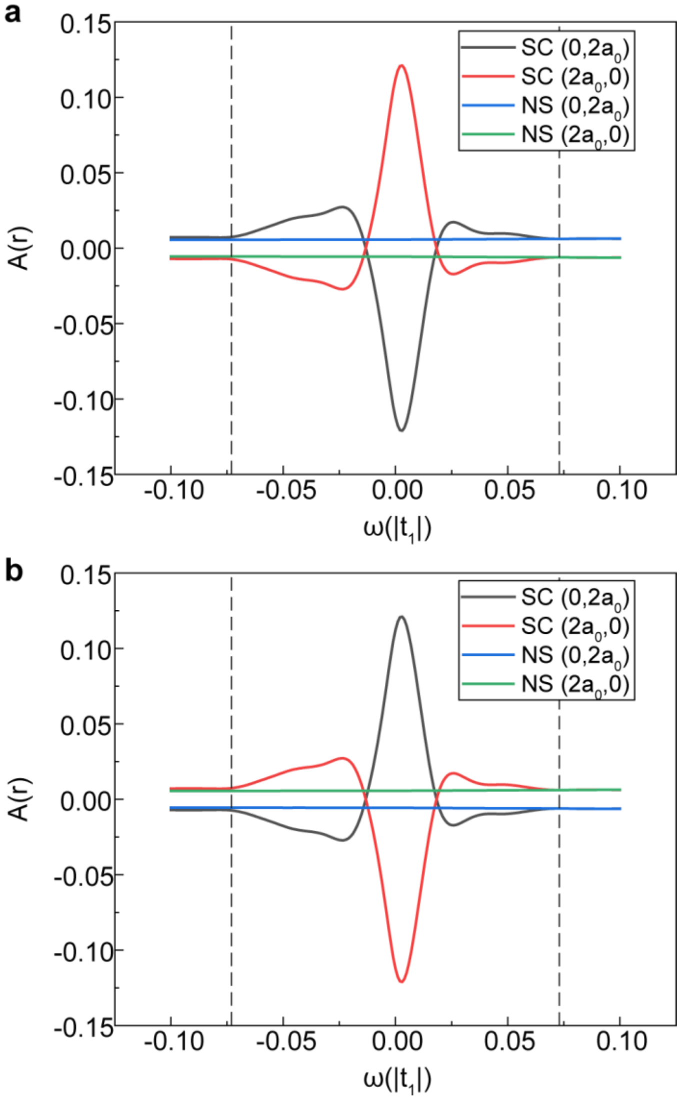

Figure S4. Local anisotropy as a function of energy along high symmetry directions (1,0) and (0,1) with the parameters described in section 1.

a,b Local anisotropy $A(\mathbf{r},\ E)$ at two sites away from the impurity along (1,0) (red curve) and (0,1) (black curve) with the impurity positioned at sublattice ***a*** (**a**) and sublattice ***b*** (**b**). Green (blue) curve is the local anisotropy $A(\mathbf{r},\ E)$ of the model in the normal state along (1,0) ((0,1)) obtained by setting $\{\Delta_1\ ,\Delta_2\} = \{0.0,\ 0.0\}$. Black dashed lines indicate the energy of superconducting gap Δ.

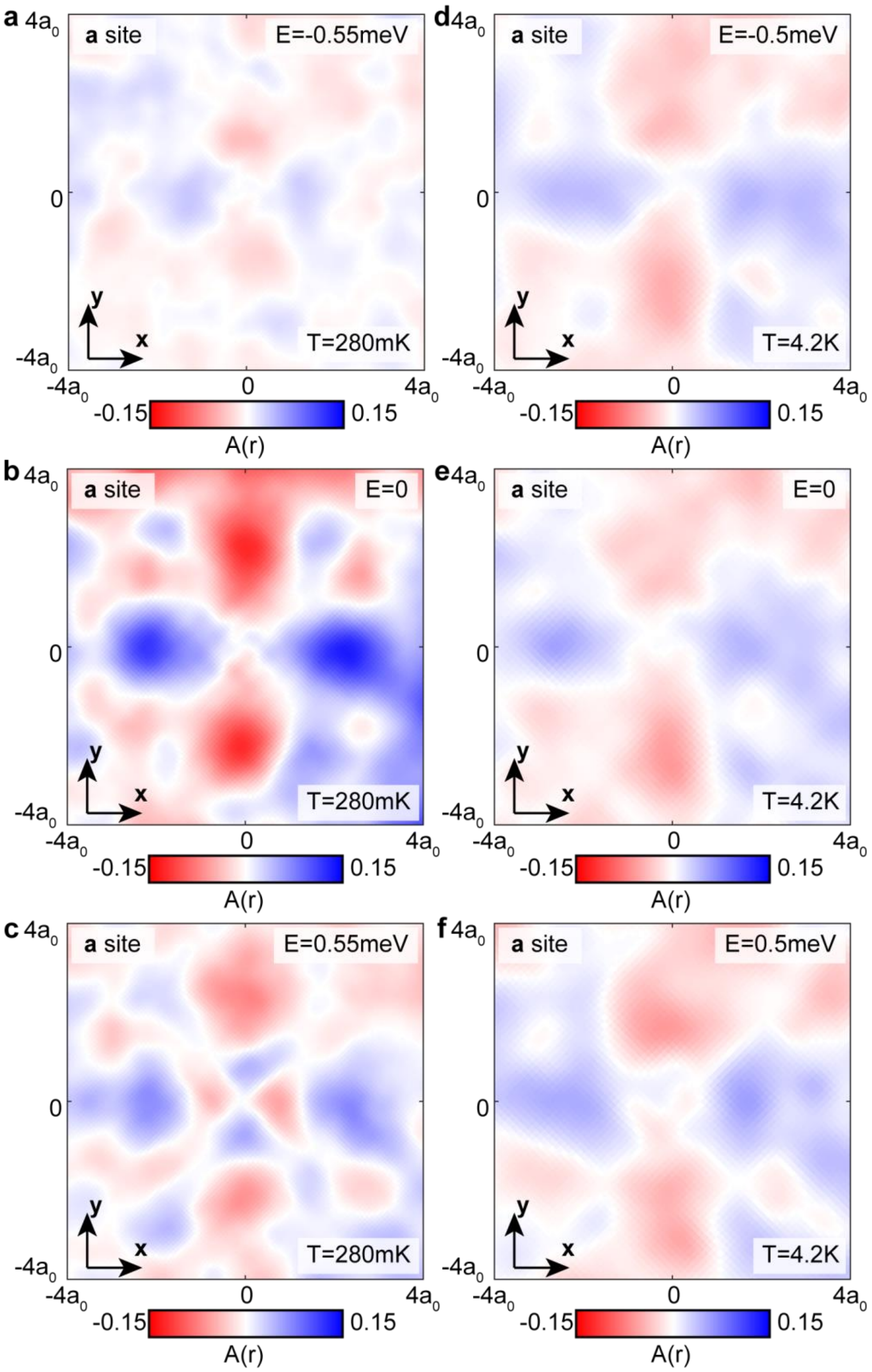

Figure S5. Local anisotropy around the same defect in superconducting state and in normal state

a,b,c Local anisotropy $A(\mathbf{r}, E)$ around the defect at sublattice $\boldsymbol{a}$ at E=-0.55 meV(**a**), E=0(**b**) and E=0.55meV(**c**) at T=280mK well below the superconducting temperature $T_c = 2.3K$.

d,e,f Local anisotropy $A(\mathbf{r},\ E)$ around the same defect in **a,b,c** at E=-0.5 meV(**a**), E=0(**b**) and E=0.5meV(**c**) at T=4.2K well above the superconducting temperature $T_c = 2.3K$.

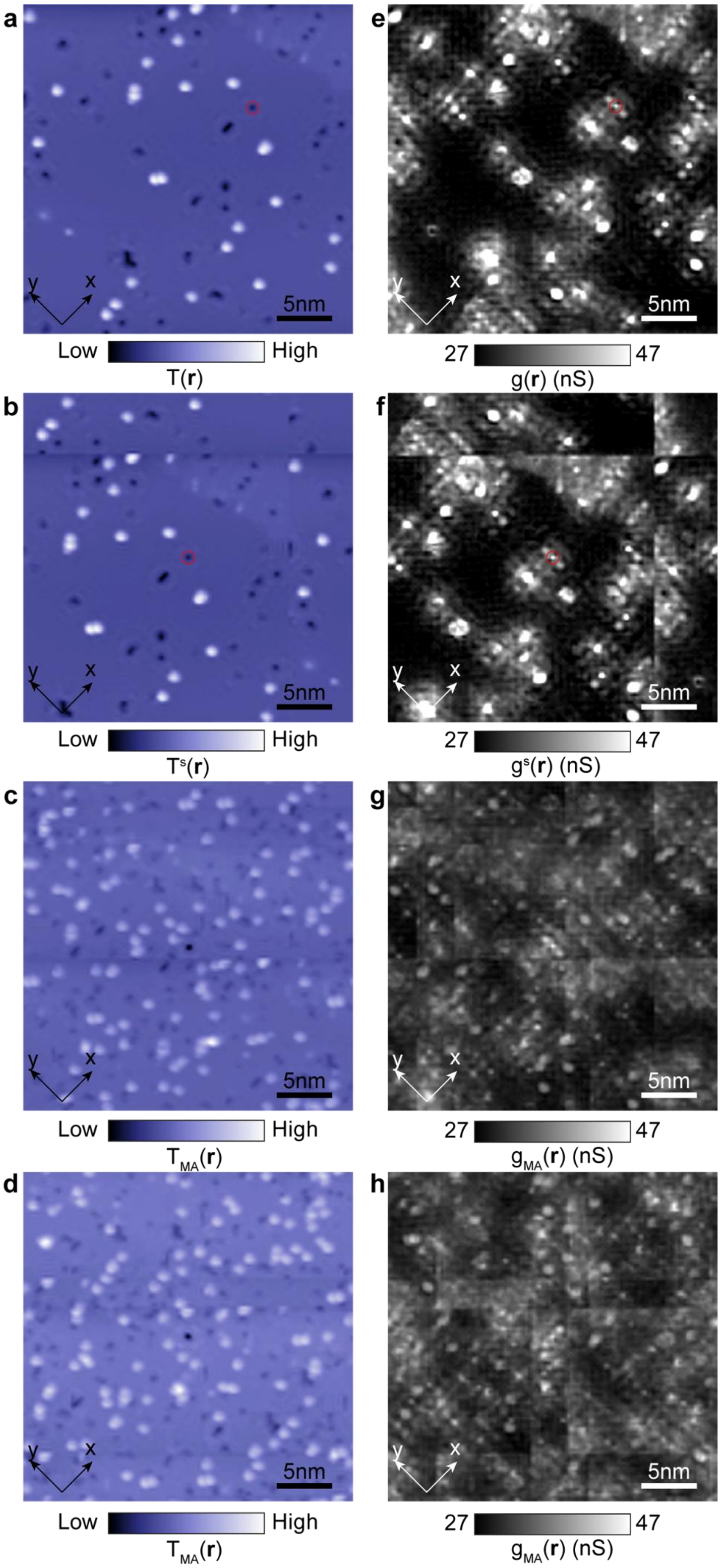
a
b
c
d
e
f
g
h
y
x
5nm
Low
High
T(r)
Tˢ(r)
T_MA(r)
27
47
g(r) (nS)
gˢ(r) (nS)
g_MA(r) (nS)

Figure S6. Multi Atom Analysis of Experimental Data in large FOV

a,e $CeCoIn_5$ topography (**a**) and $g(\mathbf{r}, E = 0)$(**e**) with a defect (shown by red circle) not at the center of the FOV.

b,f Inverse Fourier transform after applying shift theorem (Eqn. S14,S15) to the Fourier transform of a(**b**) and e(**f**) with the defect position marked by the red circle. The defect is shifted to the center of the FOV with periodic boundary conditions.

c,g The MA-averaged topography (**c**) and $g(\mathbf{r}, E = 0)$(**g**) of the defects at sublattice ***a***

d,h The MA-averaged topography (**d**) and $g(\mathbf{r}, E = 0)$(**h**) of the defects at sublattice ***b***

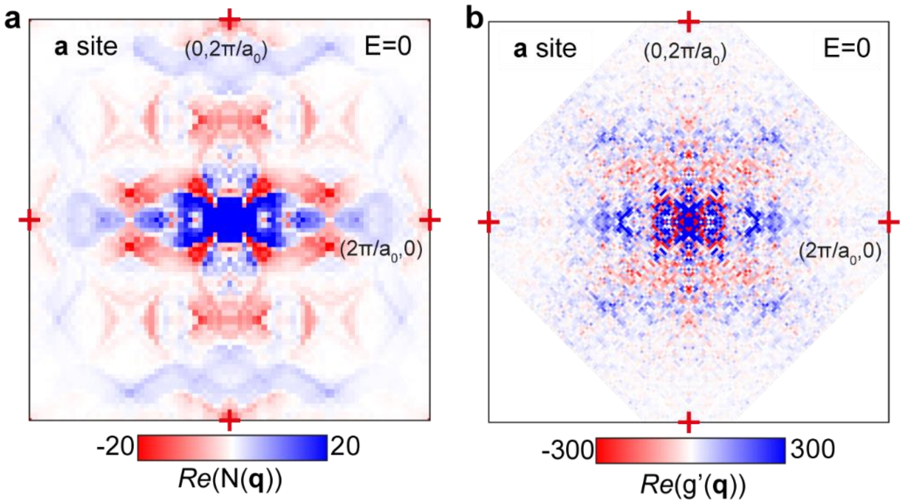

Figure S7. Fourier transformed BQPI *N(*q*,E)* at $E = 0$

a Fourier transformation of the theoretical BQPI pattern $N(\mathbf{q}, E)$ with the impurity atom at sublattice $\boldsymbol{a}$ at $E = 0$. The $\mathbf{r}$-space center of the transformation is set at the impurity site.

b Real parts of Fourier transformed MA-averaged differential conductance map $Re(g'(\mathbf{q}, E))$ at $E \sim 0$ around the defects at sublattice $\boldsymbol{a}$. Identical to Fig. 5e included here for comparison.

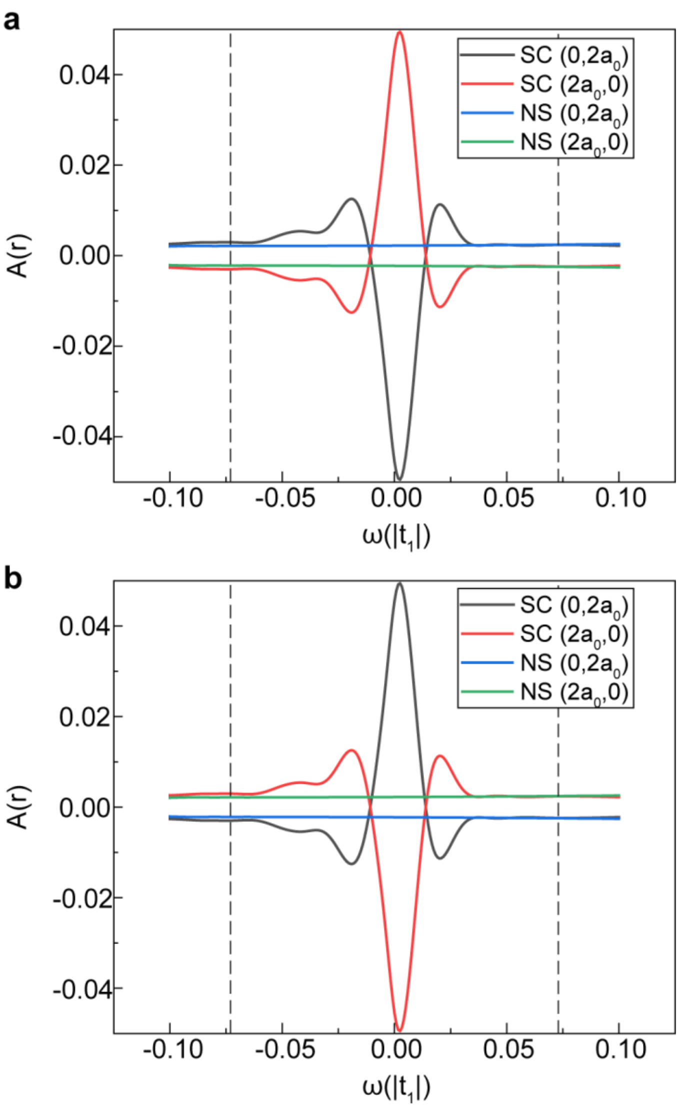

Figure S8. Local anisotropy as a function of energy along high symmetry directions (1,0) and (0,1) with $\boldsymbol{\Delta_{oo} = 0.1|t_1|}$

a,b Local anisotropy $A(\mathbf{r},\ E)$ at two sites away from the impurity along (1,0) (red curve) and (0,1) (black curve) with the impurity positioned at sublattice $\boldsymbol{a}$ (**a**) and sublattice $\boldsymbol{b}$ (**b**). Green (blue) curve is the local anisotropy $A(\mathbf{r},\ E)$ of the model in the normal state along (1,0) ((0,1)) obtained by setting $\{\Delta_1\ , \Delta_2\} = \{0.0,\ 0.0\}$. Black dashed lines indicate the energy of superconducting gap Δ.

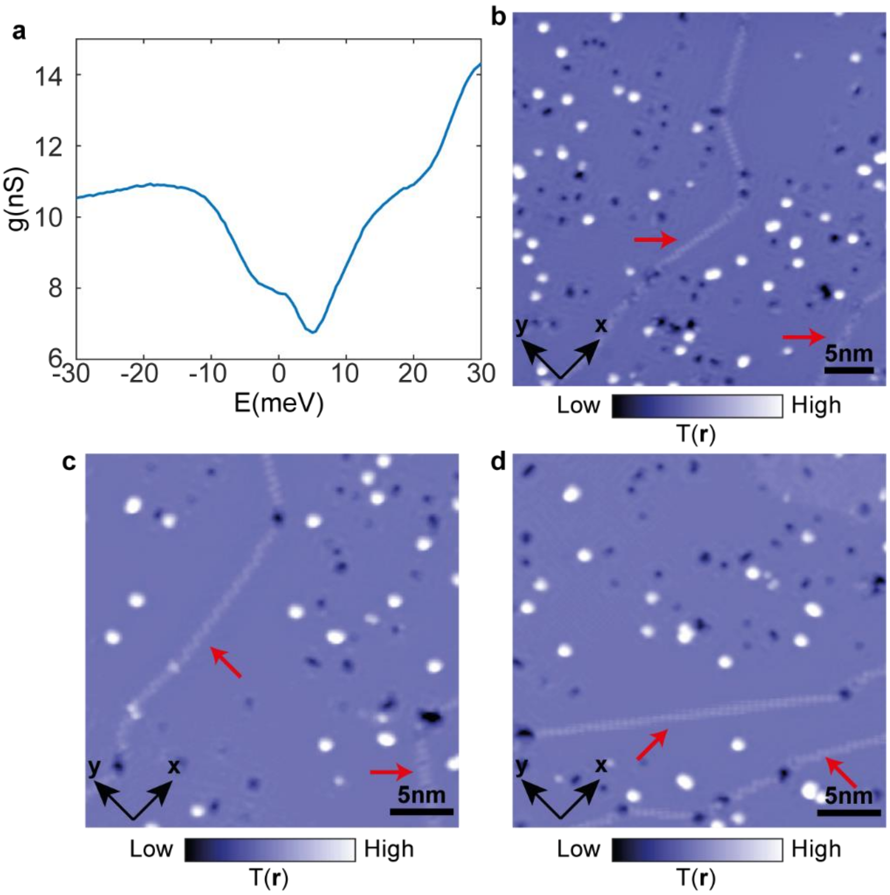

Figure S9. Determining cleaved surface

a Typical measured tunneling spectrum on cleaved surface.

b,c,d $CeCoIn_5$ topography with domain boundaries (marked by red arrows).

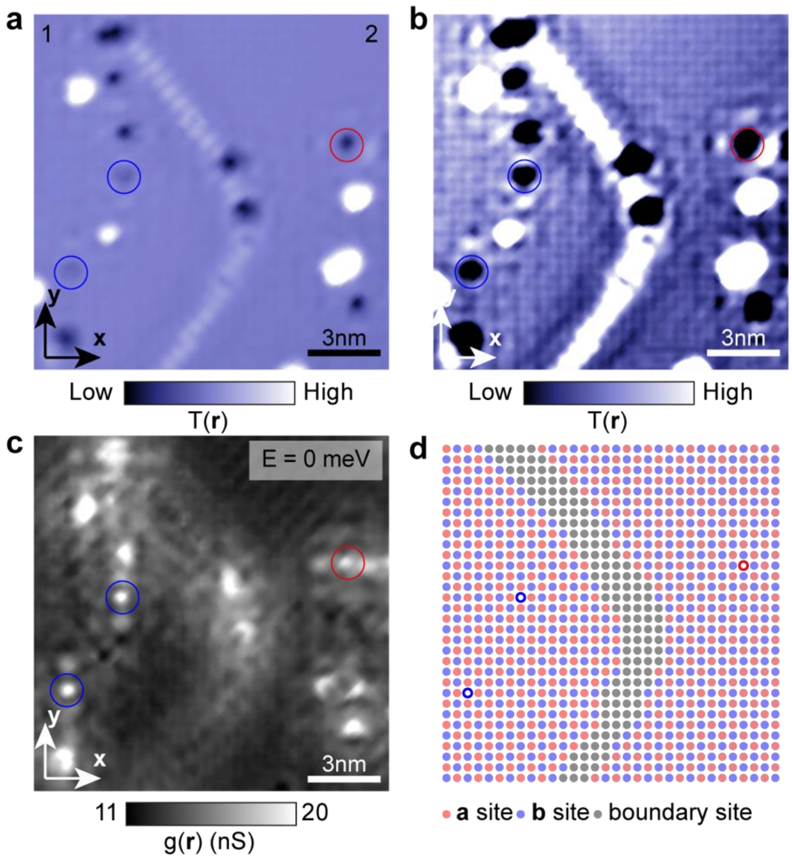

Figure S10. Orbital order domains near the domain boundary

a CeCoIn5 topography with two domains near a domain boundary.

b The same topography in **a** with adjusted colormap limit to show the atom sites.

c CeCoIn5 $g(\mathbf{r}, E = 0)$ in the same field of view in **a**.

d The schematic diagram of the arrangement of atoms with orbital order marked by red dots (***a*** site) and blue dots (***b*** site), according to **b,c**. The atoms at the domain boundary are marked by gray dots. The hollow circles show the position of the defects, corresponding to the defects marked in **a,b,c** by blue (***a*** site) or red circles (***b*** site).

References:

1 Graser, S., Maier, T. A., Hirschfeld, P. J. & Scalapino, D. J. Near-degeneracy of several pairing channels in multiorbital models for the Fe pnictides. *New Journal of Physics* **11**, 025016, doi:10.1088/1367-2630/11/2/025016 (2009).

2 Choubey, P., Berlijn, T., Kreisel, A., Cao, C. & Hirschfeld, P. J. Visualization of atomic-scale phenomena in superconductors: Application to FeSe. *Physical Review B* **90**, 134520, doi:10.1103/PhysRevB.90.134520 (2014).

3 Kreisel, A. et al. Towards a quantitative description of tunneling conductance of superconductors: Application to LiFeAs. *Physical Review B* **94**, 224518, doi:10.1103/PhysRevB.94.224518 (2016).

4 Singh, U. R. *et al.* Evidence for orbital order and its relation to superconductivity in $FeSe_{0.4}Te_{0.6}$. *Sci Adv* **1**, e1500206, doi:10.1126/sciadv.1500206 (2015).

5 Sharma, R. et al. Multi-atom quasiparticle scattering interference for superconductor energy-gap symmetry determination. *Npj Quantum Materials* **6**, 7, doi:10.1038/s41535-020-00303-4 (2021).

6 Aynajian, P. et al. Visualizing heavy fermions emerging in a quantum critical Kondo lattice. *Nature* **486**, 201-206, doi:10.1038/nature11204 (2012).

7 Kim, H. et al. Atomic-scale visualization of surface-assisted orbital order. *Science Advances* **3**, eaao0362, doi:10.1126/sciadv.aao0362 (2017).
